# Geostatistical Analysis Methods for Estimation of Environmental Data Homogeneity

**DOI:** 10.1155/2018/7424818

**Published:** 2018-06-03

**Authors:** Aleksandr Danilov, Inna Pivovarova, Svetlana Krotova

**Affiliations:** ^1^Geoecology Department, Saint-Petersburg Mining University, St. Petersburg 199106, Russia; ^2^Department of Informatics and Computer Technology, Saint-Petersburg Mining University, St. Petersburg 199106, Russia

## Abstract

The methodology for assessing the spatial homogeneity of ecosystems with the possibility of subsequent zoning of territories in terms of the degree of disturbance of the environment is considered in the study. The degree of pollution of the water body was reconstructed on the basis of hydrochemical monitoring data and information on the level of the technogenic load in one year. As a result, the greatest environmental stress zones were isolated and correct zoning using geostatistical analysis techniques was proved. Mathematical algorithm computing system was implemented in an object-oriented programming C #. A software application has been obtained that allows quickly assessing the scale and spatial localization of pollution during the initial analysis of the environmental situation.

## 1. Introduction

The construction of functional models of geosystems and prediction of the geosystems behavior are mandatory and necessary in conditions of increasing anthropogenic load in the present period of environmental studies. Optimization of nature management is an urgent need. To optimize the environmental management it is necessary to know how geosystem exists or existed in the absence of anthropogenic impact, which components of geosystems are most susceptible to anthropogenic impact, and ecosystems specificity of functioning under anthropogenic load. Classification of objects is necessary by the degree of environmental disturbance and environmental safety. Obviously, that it is wrong to apply uniform methods and standards to all geosystems. However, it is impossible to develop methods and standards for each individual geosystems because of the unacceptable time and material cost. Therefore, the necessity to integrate geosystem into taxa occurs. Application of unified methods for determining the permissible limits of anthropogenic impact and predicting their evolution is fully justified within the taxa. Thus, the task of evaluating the spatial homogeneity of ecosystems with the possibility of subsequent zoning occurs.

Environmental scientists of various countries have long paid attention to the concept of ecological zoning. In 1967, Crowley first presented the concept of ecoregion, which refers to the land and water areas with similar ecosystem or being supposed to play similar functions [1]. Basing on this concept, the purpose of ecological regionalization is to provide suitable spatial units for studying, evaluating, restoring, and managing the ecosystem [2]. The concept of aquatic ecoregion originated from America. It refers to the freshwater ecosystem or living organism and the interrelated land units [3]. The aquatic ecosystems regionalization is one of the most important fields of ecological regionalization, and it is also the field most successfully studied [4].

The “quality” of regionalization or the correspondence of allocated area to the set goals depends largely on the choice of research method. The most widely used methods of regression [5] and cluster [6, 7] analysis in many examples reveal a high degree of subjectivity. So, applying different data sets, overly careful accounting or vice versa, neglect of the influence of constantly changing anthropogenic factors can lead to different zoning schemes. Applying the methods of geostatistical analysis and cartographic visualization is most suitable for environmental problems studying (that have a pronounced spatial aspect) relying on the experience of previous research in this field [8, 9].

## 2. Materials and Methods

An attempt to reconstruct the contamination of a water body and to isolate the zones of the greatest environmental stress based on the values measured at a limited number of points was the main objective of this study. Proof of correctness zoning with certain statistical algorithms also has been a major purpose. The whole course of research was conditionally divided into two stages. The first stage is the construction of a general view, analysis, and visualization of primary data. The second stage is the use of statistical calculation methods for estimating the spatial homogeneity of environmental characteristics and finalizing the model with further software implementation.

### 2.1. The First Stage Is the Construction of a Probability Model for the Distribution of the Characteristics

This problem was considered as a problem of interpolation in mathematics. In the standard approach, an unknown function is approximated by a parametric function whose form is given either explicitly (polynomial) or implicitly (the minimum curvature condition). The parameters are chosen to optimize some criterion of best approximation values at the points. The criterion can be statistical (least squares) or deterministic (exact coincidence at measurement points). Most of the existing interpolation methods are built into modern GIS packages. The main ones are as follows [10]:

- IDW method is inversely weighted distances (average values of neighboring pixels by a predetermined number of neighbors or within a specified radius);
- Kriging is multistage selection of a mathematical function for a given number of points or for points within a given radius for propagation of dependencies on all points;
- Natural Neighbor finds the closest subset of input samples to the requested point and applies a weighted value based on proportionate areas to interpolate a value;
- Bilinear is bilinear interpolation, when the point value in the new image is calculated by linear interpolation between the values of the four nearest points;
- TIN is a method when all the starting points are connected by triangles, resulting in an irregular triangulation network.

The Mapinfo-GIS package served as a tool for building the base map in this research project.

**Figure 1:**
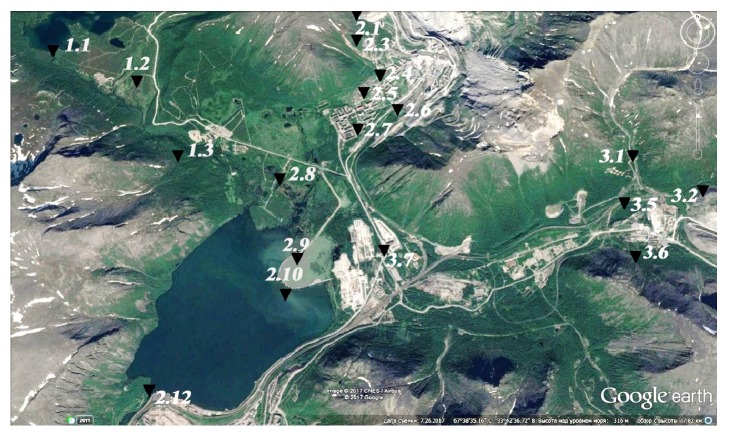
Region of research and sampling points.

The initial data for modeling were materials of ecological and hydrochemical monitoring of the state of surface water bodies and information on the level of anthropogenic impact within one year (January-December 2016) on the territory of the Khibiny mountain massif (Figure 1), located in the central part of the Kola Peninsula of the Russian Federation (Apatity mining agglomeration). The values of the content of sulfate ion (SO_4_^2−^) in surface waters were used to estimate the intensity of contamination. The probable source of sulfate ion entering the surface waters was the intensification of the extraction of the apatite-nepheline ore of the Rasvumchor field [11].

The method of inversely weighted distances (IDW) was chosen as the method of interpolation.

In the inverse distance weighted method (IDW), which can be assigned to a group of kriging methods, estimated points are determined on the basis of source points, found in its surroundings. The result is affected by several parameters such as range searches, the number of points involved in the analysis, and power factor. The process of IDW interpolation can be divided into the following steps [12]:

(1) Searching for points that meet the criterion of neighborhood (the amount or the distance).
(2) Allocating weights to each typed point. At this step, it is possible to determine the power factor (*p*); the bigger it is, the points which are farther will have a greater impact on the result.
(3) Calculating the value of estimating points [13].
  (1) wwnn=xn−x02+yn−y02−p/2zz=∑wwnn∗zznn∑wwnn,

where *w*_*n*_ is weight of the points used to interpolate, *z*_*n*_ is value of the points used in interpolation, *x*_0_, *y*_0_ are the coordinates of estimating point, *p* is power factor, and *z* is value of the estimated point.

**Figure 2:**
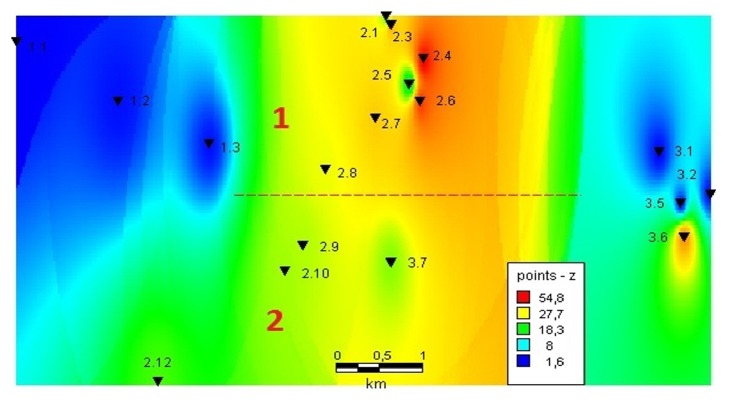
GIS-distribution of pollutants.

The method worked well with a large amount of initial data and showed the result in a convenient form for perception (Figure 2).

As a result, it was clarified that pollution is absent at points 1.1, 1.2, 1.3, 3.1, 3.2, and 3.5. Values at points 2.2, 2.11, 3.3, and 3.4 are excluded from further calculations due to data being uninformative. This is caused by close proximity. A single impregnation was detected at point 3.6, which can be caused by the infiltration of polluting components from the tailing dump of the mining enterprise located in the source of the stream. The site limited by points 2.1 and 2.12 is contaminated. The site is conditionally divided by the degree of pollution into districts I and II. The method of statistical estimation of data homogeneity was used to verify the actual presence of a spatial trend.

### 2.2. The Second Stage Is the Statistical Evaluation of the Spatial Homogeneity of Environmental Characteristics

There are a number of criteria for verifying spatial data for homogeneity. These criteria allow us to determine whether two samples (data on two different objects) are related to one general population or not [14]. If the samples belong to the same population, then the difference between the samples is within the limits of random variations of the quantities and there are no fundamental differences between the objects. In this case, parametric criteria require that the distribution of the sample is subject to a specific distribution law. Thus, the classical criteria of Student and Fisher require that the law of distribution of samples be sufficiently close to the normal law [15].* Parametric* criteria allow us to directly estimate the level of the main parameters of the general populations, the difference in the means and the difference in variances. The criteria can identify trends in data changes and evaluate the interaction of two or more factors. Recently, the Cramer and Welch criteria [16, 17] have also been used to estimate the homogeneity of data. An additional advantage of these criteria is the optional equality of the variances of the compared samples. Parametric criteria are considered to be more powerful than nonparametric ones, provided that the characteristics are measured in an interval scale and are normally distributed.

*Nonparametric* criteria do not have the above limitations. The term “nonparametric method” means that it is not necessary to assume that the distribution functions of the results of observations belong to any particular parametric group while it is used. Nonparametric criteria do not impose conditions for the recognition of the distribution law. However, criteria of this type do not allow a direct assessment of the level of such important parameters as the average or variance. Using nonparametric criteria is impossible to estimate the interaction of two or more conditions or factors affecting the change in characteristics. Many nonparametric methods have been developed, Smirnov's criteria [18], such as the omega-square (Leman-Rosenblatt) [19, 20], Wilcoxon (Mann–Whitney) [21, 22], van der Waerden [23], Savage, etc.

In addition, the affinity between the variables is usually investigated using correlation functions [24]. In this study, the calculation technique was reduced to the construction and further analysis of the homogeneity of the space-correlation function. The analysis of the function homogeneity was carried out based on the principle of assessing the significance of the difference between the actual correlation coefficient and the assumed coefficient in the total population. The Z-Fisher distribution was used as the evaluation criterion. The value of statistics obtained for the compared data groups was compared with the theoretical value at the accepted level of significance. Mathematical algorithm was as follows.

The auxiliary values were determined by the Fisher method from the values of the empirical $\tilde{r}\left(\alpha_{jk}\right)$ and theoretical *r*(*α*_*jk*_) correlation functions

(2) zjk=12ln⁡1+rαjk1−rαjkz~jk=z~αjk=12ln⁡1+r~αjk1−r~αjk+rαjk2Njk−1

and the deviation or difference $z_{jk}-\tilde{z}\left(\alpha_{jk}\right)$ was calculated for all *c*_*l*_^2^ = *l*(*l* − 1)/2 pair wise distances *α*_*jk*_ between the observation points.

Standard deviations *σ*_*zjk*_ of auxiliary variables *z*_*jk*_ from their conditional average values $\tilde{z}(\alpha_{jk})$ were determined from the formula

(3) $$\begin{array}{c} \sigma_{zjk}=\frac{1}{\sqrt{N_{jk}-1}} \end{array}$$

According to the law of normal distribution of the normalized deviations from the average value in the confidence limits

(4) $$\begin{array}{c} \tilde{z}\left(\;\alpha_{jk}\;\right)-t_{\sigma jk}<z_{jk}<\tilde{z}\left(\;\alpha_{jk}\;\right)+t_{\sigma zjk} \end{array}$$

of all *c*_*l*_^2^ = *l*(*l* − 1)/2 empirical values *z*_*jk*_ should fall *P*(*I*) = 0.683 = 68.3% for *t* = 1 or *P*(*II*) = 0.954 = 95.4% for *t* = 2.

Therefore, a necessary and practically sufficient condition for the homogeneity of the correlation function within the region under consideration is the fulfillment of inequalities

(5) $$\begin{array}{c} \left|\;z_{jk}-\tilde{z}\left(\;\alpha_{jk}\;\right)\geq \sigma_{zjk}\;\right|\text{ or}\geq 2\sigma_{zjk} \end{array}$$

approximately 31.7% or 4.6% of the total number *c*_*l*_^2^ = *l*(*l* − 1)/2 of empirical values *z*_*jk*_. In other words, for *t* = 1 and *t* = 2, the total empirical number of excesses

(6) Kэ1=Кэzjk−z~jk>σzjkKэ1=Кэzjk−z~jk>2σzjk

should be approximately equal to the theoretically possible according to the normal distribution law, the number of excesses, i.e.,

(7) Кэ1≈0,317C2l=0,317ll−12Кэ2≈0,317C2l=0,046ll−12

**Figure 3:**
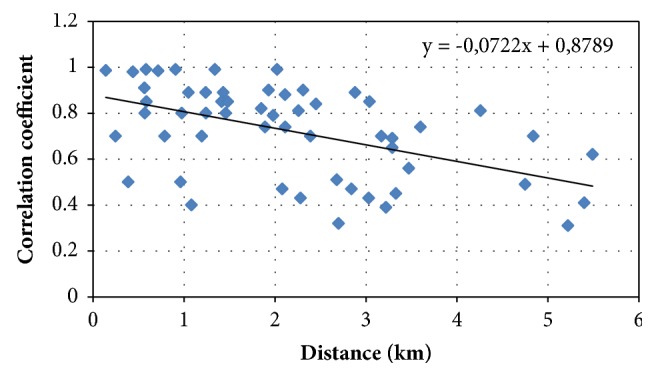
Spatial-correlation function.

Thus, the *C*_11_^2^ = (11*∗*10)/2 = 55 pairs of correlation ratios between the arrays of initial data at the site limited by points 2.1 and 2.12 were calculated. A graph of correlation dependence was constructed and the equations of theoretical and empirical correlation functions were obtained (Figure 3).

The auxiliary values of *z*_*jk*_ and $\tilde{z}(\alpha_{jk})$ were calculated from them. The standard deviation *σ*_*zjk*_ of the auxiliary values *z*_*jk*_ from their conditional average values $\tilde{z}(\alpha_{jk})$ was determined (Table 1).

As a result, it was concluded that the spatially correlation function of the region under study is inhomogeneous, since the total empirical number of exceedances is greater than the theoretically possible.

When calculating the RMS, the number of exceedances was 20, and according to the law of normal distribution, there should be 17 by 2*σ*_*jk*_: 7 and 2, respectively.

## 3. Results and Discussion

The heterogeneity of spatially distributed data within the initial study area is caused by an increased level of technogenic impact along the line of sampling points 2.1–2.12. Discharges of sewage from a mining enterprise are located in the catchment basin of small rivers in this area.

The remaining sampling points are located on small rivers and streams, the catchment basins of which lie at the base of the Khibiny mountain massif and the main power source is the melting of snow in the summer season and precipitation; this causes a low content of polluting components, including sulfate.

In order to obtain a conclusion on the zoning of ecological components, this region is divided into two subareas: 1, 2 (determined by the results of the previously constructed GIS project) for each of which similar calculations were made, and a conclusion was made about the homogeneity of environmental characteristics in each of them.

The preliminary carried out reconnaissance studies indirectly point to correctness of the proposed results. [25]. Sampling points 1.1–1.3 are located in the zone of the botanical garden of the Kola Branch of the Russian Academy of Sciences and are not affected by any technogenic impact. In the upper course of the river, in the area of point 2.4, a discharge of wastewater from a mining and processing plant with an extremely high content of sulfate ion in water has been detected. At the same time, there are no large tributaries in the investigated area. For this reason, the sewage hardly changes its composition and the uniformity of ecological parameters throughout the site (region 1). After sampling point 2.8, there is a mixture of pure natural waters from the Khibin foothills with industrial wastewater. Also, a large volume of water flows of meltwater enters the river system. Thus, in region 2, there is a general decrease in the concentration of polluting components due to dilution of sewage waters of the mining enterprise with clean natural waters, and because of the absence of inflows downstream this area is defined as ecologically homogeneous.

### 3.1. Finalization of the Methodology and Automation of Calculations

Numerous calculations and a large amount of input data have revealed the necessity to develop a software to solve the task, despite the good results of using the methodology. It was decided to replace the segment of the graphic finding of the parameters of the equation of the empirical and theoretical correlation functions by the construction of approximating dependencies. In the future, the approximation parameters were found from the condition of a minimum of the total quadratic error (least squares method) in order to fully automate the whole process of calculations from the introduction of the initial data to obtaining a response about the homogeneity of the characteristics studied. Thus, the parameters of the equation *y* = *ax* + *b* form were found by the formulas

(8) a=n∑i=1nxiyi−∑i=1nxi∑i=1nyin∑i=1nxi2−∑i=1nxi2b=∑i=1nyi−a∑i=1nxin,

where *n* is the number of terms in the series, *x*_*i*_ is the distance between the observation points, and *y*_*i*_ is the pairwise correlation coefficient. Further calculations were made according to the above algorithm.

**Figure 4:**
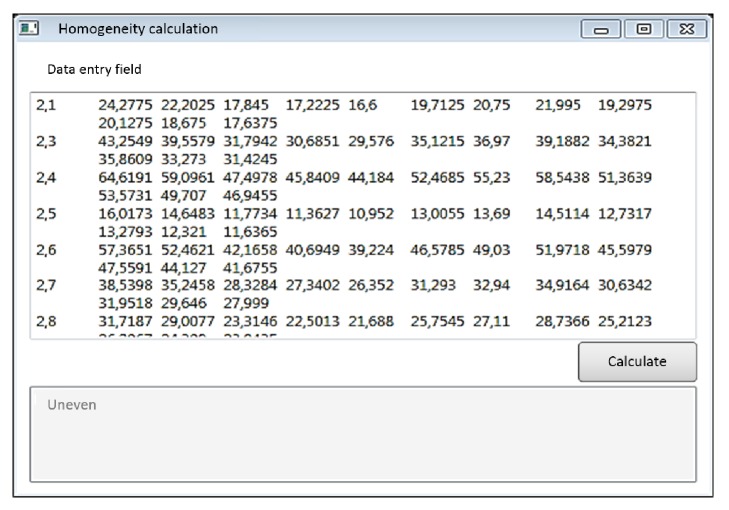
Example of calculations in the software.

The result was the implementation of a mathematical computation algorithm in the system of object-oriented programming C # (Figure 4). Simplicity of use, full compatibility with Windows and all office applications, loading of initial data from MS Excel, and being undemanding to a certain format of initial data make the developed software solution a convenient tool for the user.

The main result is that the software allow any ecologist to quickly assess the scale and spatial localization of pollution at the stage of the initial analysis of the environmental situation.

## 4. Conclusion

The process of constructing a model of the spatial structure of various natural systems is quite complex and requires the joint consideration of a large number of very diverse factors. This heterogeneity itself has both a thematic and a spatial nature. The spatial heterogeneity of information is expressed in the fact that statistical and descriptive data are often correlated with different spatial objects that differ in nature and in scale, which creates additional difficulties in the joint processing and analysis of information. Therefore, in problems of this kind, the role of coordinate data binding is great, without which spatial analysis does not make sense. Pollution zones are geographically related to sources of environmental hazard. The strength of the hazardous effect and possible damage depend on the proximity of the risk element to the source of contamination, and the risk depends on the frequency of the dangerous manifestations. Thus, when allocating zones of adverse impact, the use of a geographic coordinate space is necessary to assess the area and intensity of environmental damage. That was done in our work at the first stage of research. Moreover, since the basic sample map showed the possible presence of a spatial trend in the data, this fact was verified by statistical methods. For this purpose, the relationship between the values of the investigated variable and coordinates in a two-dimensional space is distinguished using various indicators of the correlation relationship. The result of the work is the development and successful use of a certain mathematical algorithm with its further software solution for estimating the uniformity of spatially distributed data. Creation of the information model of the investigated territory is reflecting the spatial structure and location of the zones of environmental pollution.

## Figures and Tables

**Table 1:** Estimation of homogeneity of cross-correlation function of the investigated descriptions (a fragment over of calculations is brought on 12 pairs from 55).

Pairs	Distance between sampling points	Coefficient of pair wise correlation	Value of empiric cross-correlation function	Value of theoretical cross-correlation function	*Z*_*jk*_	${\tilde{Z}}_{jk}$	Rejection (Δ)	Mean square error (*δ*)	Doubled mean square error (2*δ*)	Case, when a rejection exceeds a MSE (Δ > *δ*)	Case, when a rejection exceeds a doubled MSE (Δ > 2*δ*)
2,1-2,3	0,14	0,986	0,868792	0,988499	2,576362	1,373064	1,203298	0,301511	0,603023	1	1
2,1-2,4	0,716	0,984	0,827205	0,941182	1,748302	1,221999	0,526303	0,301511	0,603023	1	
2,1-2,5	0,963	0,5	0,809371	0,920891	1,594861	1,167063	0,427799	0,301511	0,603023	1	
2,1-2,6	1,196	0,7	0,792549	0,901751	1,481512	1,119238	0,362274	0,301511	0,603023	1	
2,1-2,7	1,34	0,99	0,782152	0,889921	1,421548	1,091341	0,330208	0,301511	0,603023	1	
2,1-2,8	2,114	0,74	0,726269	0,826339	1,176481	0,958347	0,218134	0,301511	0,603023		
2,3-2,4	0,587	0,85	0,836519	0,951779	1,850351	1,252727	0,597625	0,301511	0,603023	1	
2,3-2,5	0,789	0,7	0,821934	0,935185	1,698212	1,205259	0,492953	0,301511	0,603023	1	
2,3-2,6	1,083	0,4	0,800707	0,911034	1,53357	1,141991	0,391579	0,301511	0,603023	1	
2,3-2,7	1,241	0,8	0,7893	0,898054	1,462071	1,110392	0,351679	0,301511	0,603023	1	
2,3-2,8	2,024	0,99	0,732767	0,833732	1,200254	0,972574	0,22768	0,301511	0,603023		
2,4-2,5	0,387	0,5	0,850959	0,968209	2,062843	1,303627	0,759216	0,301511	0,603023	1	1

## Data Availability

The data used to support the findings of this study are available from the corresponding author upon request.
